# Computer‐aided prediction of growth in vestibular schwannomas based on both structural and dynamic contrast‐enhanced MR imaging

**DOI:** 10.1002/mp.70224

**Published:** 2025-12-29

**Authors:** Stefan Cornelissen, Sammy M. Schouten, Daniel Lewis, Ka‐Loh Li, Xiaoping Zhu, Marnix C. Maas, Sjoert Pegge, Thijs T. G. Jansen, Jef J. S. Mulder, Patrick P. J. H. Langenhuizen, Andrew T. King, Jeroen B. Verheul, Henricus P. M. Kunst, Peter H. N. De With

**Affiliations:** ^1^ Gamma Knife Center Department of Neurosurgery Elisabeth‐TweeSteden Hospital Tilburg The Netherlands; ^2^ Department of Electrical Engineering Eindhoven University of Technology Eindhoven The Netherlands; ^3^ Department of Otolaryngology Radboud University Medical Center Nijmegen The Netherlands; ^4^ Department of Otolaryngology Maastricht University Medical Centre+ Maastricht The Netherlands; ^5^ Dutch Academic Alliance Skull Base Pathology Radboud University Medical Center/Maastricht University Medical Centre+ Nijmegen/Maastricht The Netherlands; ^6^ Mental Health & Neuroscience Research Institute Maastricht University Maastricht The Netherlands; ^7^ Geoffrey Jefferson Brain Research Centre University of Manchester Manchester UK; ^8^ Division of Cancer Sciences University of Manchester Manchester UK; ^9^ Department of Medical Imaging Radboud University Medical Center Nijmegen The Netherlands

**Keywords:** dynamic contrast‐enhanced, prediction model, vestibular schwannoma

## Abstract

**Background:**

Vestibular schwannomas (VS) are benign intracranial tumors in the cerebellopontine angle. For most small‐sized and medium‐sized tumors, a wait‐and‐scan (W&S) approach is employed, since active treatment does not necessarily lead to symptom alleviation and up to 60% of VS tumors remain stable or regress during their natural course. At diagnosis, it is currently not possible to reliably predict tumor behavior and whether the tumor will grow or not, although some studies employing dynamic contrast‐enhanced (DCE) magnetic resonance imaging (MRI) or artificial intelligence (AI) suggest potential evidence.

**Purpose:**

Personalized clinical decision‐making for VS patients may be improved if tumor growth can be predicted. This study therefore prospectively investigates the use of both structural T2‐weighted MRI and DCE‐derived microvascular biomarker maps (K^trans^, v_e_, and v_p_) in combination with machine learning techniques for the short‐term prognosis of VS.

**Methods:**

Newly diagnosed patients with unilateral sporadic VS and considered for an initial W&S strategy were enrolled between January 2021 and January 2023. Study participants underwent MR imaging, including both T2‐weighted and DCE‐imaging, within 3–6 months after diagnosis and were followed‐up according to the usual standard‐of‐care regimen. The DCE‐derived parameter maps (K^trans^, v_e_, and v_p_) were calculated using established pipelines. Radiomic features are extracted from the structural MR images and the three DCE‐derived parameter maps. The resulting feature vector is reduced in dimensionality, using an *F* test and principal component analysis (PCA). A support vector machine (SVM) model is subsequently trained for the classification of tumor growth. The results of the *F* test are employed to assess the predictive value of each calculated feature.

**Results:**

A total of 110 patients are analyzed for this study, of which 70 (64%) exhibited growth during follow‐up. After fivefold cross‐validation, the SVM model yields a mean accuracy of 89.0%, sensitivity of 90.0%, specificity of 87.7%, and AUC of 0.89 at the optimized cut‐off probability for the prediction of tumor growth. The K^trans^ and v_e_ parameter maps provide most of the features to the model, comprising mostly of complex radiomics, as opposed to first‐order statistics.

**Conclusions:**

The developed model demonstrates the high potential of the combination of machine learning techniques and dynamic MRI for the prediction of tumor growth in VS patients. The DCE‐derived parameters show high predictive value and provide insight into possible links between tumor biology and growth mechanisms. Complex radiomic features have revealed to be superior to first‐order statistics as predictors for VS tumor growth, while preserving a degree of model explainability. Prior to clinical implementation, the reproducibility of the radiomics and model itself should be externally validated.

## 1 INTRODUCTION

Vestibular schwannomas (VS) are benign intracranial tumors that arise from the vestibulocochlear nerve in the cerebellopontine angle. They have an estimated lifetime prevalence in excess of 1:500 individuals, and commonly present with sensorineural hearing loss, tinnitus, and/or vestibular symptoms.^1^ A wait‐and‐scan (W&S) approach is increasingly employed for small‐sized and medium‐sized tumors, since focus of tumor management has shifted from upfront treatment with either surgical resection or radiosurgery to more conservative strategies.^2^, ^3^, ^4^ This shift is partially instigated by new clinical insights from the last decades that upfront treatment may not necessarily lead to symptom alleviation, specifically with regard to improving audiological outcome over the natural history in the long‐term.^5^, ^6^, ^7^ Equally important, up to 60% of VS tumors remain stable or even regress during their natural course.^2^, ^3^, ^4^, ^8^, ^9^ However, an active treatment strategy such as microsurgery, stereotactic radiosurgery (SRS), or a combination of both, is usually preferred when tumor growth is observed to forestall symptom progression due to an increased mass effect on adjacent anatomical structures such as the brainstem and surrounding cranial nerves.^2^, ^10^

The ability to predict tumor growth for VS patients assigned to a W&S strategy would improve personalized clinical decision‐making. If growth could be reliably predicted on the initial diagnostic scan diagnosis, patients with anticipated growth would be offered earlier intervention that may result in improved clinical outcomes, while other patients may be monitored reliably with less frequency.^11^ Generally, VS are considered to be unpredictable in nature.^2^ However, an increasing number of studies provide some evidence that VS tumor growth can be predicted, based on clinical variables or imaging data. Unfortunately, no fully reliable and conclusive variable or parameter to predict growth has been identified up to now.^11^, ^12^, ^13^, ^14^, ^15^, ^16^

Recently, several pilot studies have demonstrated the potential value of advanced magnetic resonance imaging (MRI) techniques such as dynamic contrast‐enhanced (DCE) MRI, to provide information on intrinsic VS tumor biology.^17^, ^18^, ^19^, ^20^ This technique allows for the evaluation of tissue microvascular features by acquiring high‐resolution imaging in the temporal domain, after the administration of a gadolinium‐based contrast agent. Through using model‐based quantification, such as the extended Tofts model (ETM),^21^ important microvascular kinetic parameters can be derived, such as K^trans^, v_e_, and v_p_. Herein, the volume transfer constant K^trans^ is a mixed parameter reflecting microvascular blood flow, vessel permeability, and vessel surface area. Furthermore, the ratio of each image voxel occupied by extracellular extravascular space and by blood plasma is represented by v_e_, and v_p_, respectively. Building upon these insights, we have recently prospectively assessed the predictive value of DCE‐MRI‐derived microvascular biomarkers for the short‐term prognosis of VS. To this end, a well‐performing logistic regression model based on first‐order statistics (FOS) of the DCE‐derived parameters has been developed, which exemplifies the high prospect of these biomarkers for the prediction of tumor growth.^14^

Alongside advanced MRI techniques, artificial intelligence (AI) combined with radiomics has demonstrated its ability to extract meaningful information from complex data (e.g. MRI) and subsequently make reliable predictions for clinical applications.^22^ Earlier studies have shown the potential value of these techniques for the prediction of VS tumor growth using structural MRI.^12^, ^16^, ^23^, ^24^, ^25^, ^26^ This study therefore investigates the use of these machine learning methods to predict short‐term tumor growth in sporadic VS, based on both DCE‐MRI‐derived parameters and structural imaging.

## 2 MATERIALS AND METHODS

### 2.1 Study population

**FIGURE 1:**
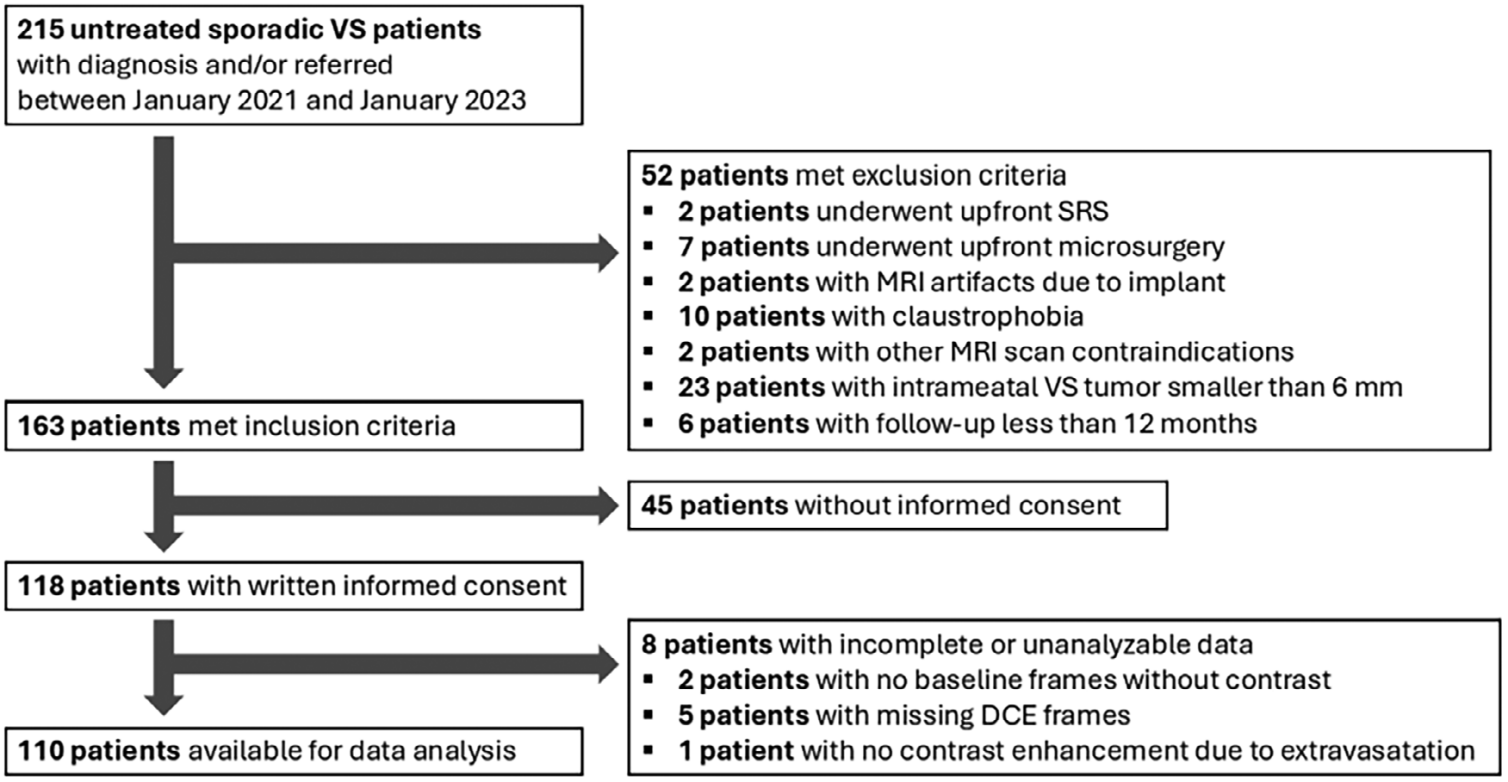
Flowchart showing the inclusion and exclusion process of the study population. DCE, dynamic contrast‐enhanced; MRI, magnetic resonance imaging; SRS, stereotactical radiosurgery; VS, vestibular schwannoma.

This study employs a subset of the previously acquired dataset by Schouten et al.^14^ Institutional review board approval was obtained for this prospective study, as well as written informed consent from all study participants. Between January 2021 and January 2023, newly diagnosed patients with unilateral sporadic VS that were considered for initial W&S strategy, were selected and invited for study participation through the Skull Base Unit at the Radboud University Medical Center Nijmegen, The Netherlands. A flowchart of the study population with applicable exclusion criteria is depicted in Figure 1.

### 2.2 Image acquisition

Participants underwent MRI within 3–6 months after diagnosis on a 3T MAGNETOM Prismafit system (Siemens Healthineers, Erlangen, Germany), employing a 20‐channel head and neck coil. The imaging protocol included thin‐slice three‐dimensional T2‐weighted imaging, precontrast quantitative T1‐mapping, and DCE imaging sequences (see Supplementary Methods 1). Participants were followed‐up according to the usual standard‐of‐care regimen using conventional high‐resolution T2‐weighted imaging at 1, 2, and 4 years after diagnosis. As per usual standard‐of‐care, participants were converted to treatment if (continued) radiographic tumor growth was detected during follow‐up.

### 2.3 Data processing

T2‐weighted images were normalized using Z‐score normalization to reduce variability across scans and mitigate adverse effects caused by data heterogeneity. The DCE‐derived parameter maps (i.e. K^trans^, v_e_, and v_p_) were derived by analysts Ka‐Loh Li, Xiaoping Zhu, and Daniel Lewis (who were blinded to tumor growth outcome) from the DCE imaging sequences, by fitting the concentration‐time curves of the contrast agent for each individual patient to the extended Tofts model (ETM)^14^ (see Supplementary Methods 2).

Tumor volume is measured for initial and follow‐up imaging by manual slice‐by‐slice annotation of the T2‐weighted images, using ITK‐SNAP (Version 3.8.0).^27^ All images are annotated by a single reader (SS) and are subsequently independently reviewed by a second reader (SC). In case of annotation disagreement, annotations are revised by the first reader until consensus between the two readers is reached. An earlier published interobserver variability study on tumor volume segmentation, performed by the same annotators, resulted in a mean inter‐rater correlation coefficient of 0.99, indicating excellent agreement between the annotators.^28^

For determining the tumor region of interest (ROI) for DCE‐derived parameter estimation, the volumetric tumor annotation on the initial T2‐weighted image is taken and cystic components are removed because they lack cellularity and microvascularity. Because of the lower spatial resolution of DCE imaging sequences and potentially resulting partial volume effects, the annotation is also morphologically eroded using a 3 × 3 cross‐shaped structural contour filter, to ensure that all voxels inside the ROI are fully captured within the tumor contour. The effects of this operation on the annotation are elaborated in Supplementary Methods 3. The resulting ROI object masks are then coregistered with a baseline frame of the dynamic DCE series to enable their projection onto the K^trans^, v_e_, and v_p_ parameter maps.

The data is labeled using a patient‐specific and volume‐dependent threshold of significant volumetric relative change. This threshold is defined as the upper bound of the limits of agreement with the mean (LOAM) for VS, as proposed by Cornelissen et al.^28^ This method provides a volume‐dependent threshold range of approximately 5%–30%. The corresponding online calculator is employed to calculate the subject‐specific relative volumetric change threshold for each patient, based on their measured tumor volume at diagnosis (https://vs‐study.shinyapps.io/loamcalculation/). If significant tumor progression or regression with respect to the tumor volume at diagnosis is identified on two consecutive follow‐up scans, tumors are accordingly labeled as either growing or shrinking. In the case of no significant change in tumor volume, cases are labeled as stable. The employed labeling method is the same as in the study by Schouten et al.,^14^ which therefore results in identical data labels. This enables a fair comparison between the two model development methods.

### 2.4 Feature extraction and model development

The processed image data acquired at diagnosis with the determined labels are employed for the subsequent development of a binary classification model that predicts either tumor growth or nongrowth (i.e. stable or regressing tumors). Because of the small size of the dataset preventing the use of modern AI techniques such as deep learning, the classical machine learning approach of support vector machines (SVM) with manual feature extraction through radiomics is employed. This approach has demonstrated its effectivity in binary classification on small VS datasets.^24^ Python (Version 3.8.8) with the scikit‐learn (Version 1.3.2^29^;) and PyRadiomics (Version 3.1.0^30^;) libraries are employed for model development and performance assessment. All non‐deterministic code used a fixed random seed of 2024 to ensure reproducibility.

Radiomic features of the voxels inside the tumor ROI are extracted from the T2‐weighted images and the DCE‐derived parameter maps. For the discretization procedure, a modality‐dependent fixed‐bin width is employed based on the mean voxel intensity range of the training set for each specific image or map, divided by the scaling hyper‐parameter *N_bins_*. No value clipping, truncation, or outlier exclusion was performed prior to discretization. Subsequently, all 101 available radiomic features in the PyRadiomics package are calculated for each image and map (i.e. T2, K^trans^, v_e_, and v_p_), comprising of 18 first‐order features, 8 shape features, 24 gray‐level cooccurrence matrix (GLCM) features, 16 gray‐level run‐length matrix (GLRLM) features, 16 gray‐level size‐zone matrix (GLSZM) features, 5 neighboring gray‐tone difference matrix (NGTDM) features, and 14 gray‐level dependence matrix (GLDM) features. This results in a feature vector of 404 elements for each individual patient. An overview of the final PyRadiomics configuration can be found in Supplementary Methods 4.

The SVM model is trained and validated using nested stratified fivefold cross‐validation. In order to mitigate any risk of model overfitting, the dimensionality of the original feature vector is reduced as follows. In each fold, (1) all features are standardized by subtracting the mean and scaled to unity variance (i.e. Z‐score normalization). (2) The top‐*k* features are determined using an *F*‐test, where the features with the *k*‐highest test statistics are selected for model development. (3) Principal component analysis (PCA) is employed to reduce the dimensionality further to *n* features (where *n *< *k*). This results in a feature vector of length *n* for each sample.

The model is subsequently trained by optimizing the following hyper‐parameters: SVM kernel type and their corresponding parameters, regularization parameter *C*, *N_bins_*, and the dimensionality reduction parameters *k* and *n*. Class imbalance effects are mitigated by using class weights. The optimal model is determined by assessing the accuracy, specificity, sensitivity, area under the receiver operating characteristic curve (AUC), and area under the precision‐recall curve (AUCPR) performance metrics. The optimal cut‐off probability is calculated for each training fold using Youden's J statistic and only applied to the corresponding validation fold. Additional performance metrics are calculated for the best‐performing model, specifically the false positive rate (FPR), false negative rate (FNR), and diagnostic odds ratio (DOR). Furthermore, two clinically relevant operating points (i.e. rule‐in and out) are assessed posthoc. The calibration of the model is subsequently assessed using calibration‐in‐the‐large, Brier score, and calibration and decision curves from out‐of‐fold samples only.

Additionally, a brief ablation analysis is conducted to assess the added value of DCE‐based radiomic features. This is achieved by training three baseline models: one solely based on the tumor volume at diagnosis, one trained exclusively on the 101 T2‐based features, and another trained solely on the 54 FOS of the DCE‐derived parameter maps. The AUC values of the baseline models are compared to the performance of the model employing the full feature set, using a paired bootstrap analysis (*N* = 1000), where *P* values lower than 0.05 are considered significant.

### 2.5 Predictive value of features

By investigating the outcome of the feature selection process, it is possible to infer what types of features contribute to the predictability of tumor progression. Since feature‐specific information is lost during the execution of the PCA, the top‐*k* features after the *F* test are analyzed. However, to increase model transparency, the permutation importance of the *n* principal components employed by the SVM is reported. First, the intersection of the top‐*k* feature sets of all five cross‐validation folds is calculated, resulting in a list of features utilized in the model development of all folds. These features are subsequently grouped by their meaning and representation, based on their definition in the ‘Image Biomarker Standardisation Initiative^31^. To exploratively assess the discriminative ability of each feature in predicting tumor growth, the effect size of each feature between the growing and non‐growing tumor groups is calculated using Cohen's *d* method.

## 3 RESULTS

### 3.1 Dataset

From the total of 118 included patients for this study, the data from 8 participants could not be analyzed (see Figure 1), resulting in a final dataset of 110 patients (57 (52%) male, median age at diagnosis 58 years (interquartile range (IQR): 48–67)). Median tumor volume is found to be 0.7 cm^3^ (IQR: 0.2–2.3) at diagnosis and 23 (21%) tumors displayed cystic components. During follow‐up, tumor regression and stability are observed in 15 (14%) and 25 (23%) tumors, respectively, resulting in a total of 40 (37%) nongrowing tumors. Growth is detected in 70 (64%) tumors, of which 50 (45%) patients are converted to active treatment (48 patients to SRS; 2 patients to microsurgery) at a median time of 15 months (IQR: 9–22). To define initial growth and its confirmation, growth was first detected at a median time of 10 months (IQR: 4–15) and confirmed during a consecutive follow‐up at a median time of 14 months (IQR: 8–21), with a median time of 6 months (IQR: 4–11) between the detection and confirming scan. Patients with nongrowing tumors have a median follow‐up time of 25 months (IQR: 17–35). Table 1 summarizes the patient and tumor characteristics.

**TABLE 1:** Patient and tumor characteristics.

Total included patients		110 (100%)
■ Growing tumors		70 (64%)
■ Nongrowing tumors		40 (37%)
○ Regression		15 (14%)
○ Stability		25 (23%)
Age at diagnosis (years)		58 (48–67)
Sex (male)		57 (52%)
Volume (cm^3^)		0.7 (0.2–2.3)
Maximum extrameatal diameter (mm)		13 (7–18)
Koos grade	1	22 (20%)
	2	30 (27%)
	3	15 (14%)
	4	43 (39%)
Cystic components		23 (21%)
■ Microcystic		15 (14%)
■ Macrocystic		8 (7%)
Number of follow‐up MRIs		3 (2‐4)
Conversion to treatment		50 (45%)
■ SRS		48 (44%)
■ Microsurgery		2 (2%)
Time to treatment (months)		15 (9–22)
Time to detected growth (months)		10 (4–15)
Time to confirmed growth (months)		14 (8–21)
Time between detection and confirming scan for growth (months)		6 (4–11)
Follow‐up time of stable and regressing tumors (months)		25 (17–35)

*Note*. Data are either presented as median with inter‐quartile range in parentheses or the number of instances with percentage of the total patients in parentheses.

Abbreviations: MRI, magnetic resonance imaging; SRS, stereotactic radiosurgery.

### 3.2 Model performance

**FIGURE 2:**
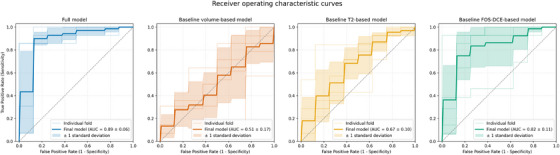
Receiver operating characteristic (ROC) curves for the developed prediction models for tumor growth: the full model and the baseline models employing tumor volume at diagnosis, T2‐based radiomics, and first‐order statistics of the DCE‐derived parameters. Results from each cross‐validation fold are shown individually, along with the mean ROC curve (i.e. the final model performance) and its ± 1 standard deviation. AUC, area under the curve; DCE, dynamic contrast‐enhanced; FOS, first‐order statistics.

**FIGURE 3:**
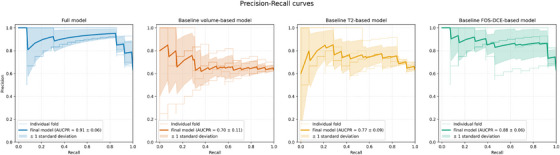
Precision‐recall curves for the developed prediction models for tumor growth: the full model and the baseline models employing tumor volume at diagnosis, T2‐based radiomics, and first‐order statistics of the DCE‐derived parameters. Results from each cross‐validation fold are shown individually, along with the mean precision‐recall curve (i.e. the final model performance) and its ±1 standard deviation. AUCPR, area under the precision‐recall curve; DCE, dynamic contrast‐enhanced; FOS, first‐order statistics.

**FIGURE 4:**
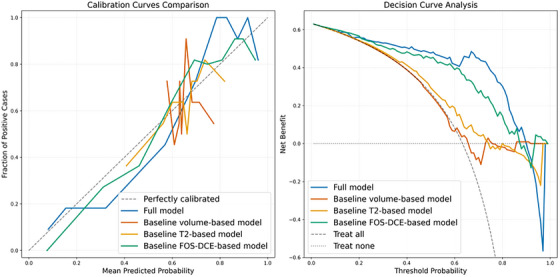
Calibration and decision curves for the developed models for tumor growth: the full model and the baseline models employing tumor volume at diagnosis, T2‐based radiomics, and first‐order statistics of the DCE‐derived parameters. DCE = dynamic contrast‐enhanced; FOS, first‐order statistics.

After hyper‐parameter tuning, the best‐performing model is found to be an SVM with a Gaussian kernel (*N_bins_ *= 32, *k *= 100, *n* = 25, variance retained by PCA = 98.9 ± 0.1%). This model yields a mean AUC of 0.89 ± 0.06 and AUCPR of 0.91 ± 0.06, with a mean accuracy of 89.0 ± 4.0%, sensitivity of 90.0 ± 6.6%, and specificity of 87.7 ± 8.8% at the optimal cut‐off probability of 0.625 ± 0.066. This corresponds to a false positive rate (FPR) of 12.3%, false negative rate (FNR) of 10.0%, and diagnostic odds ratio (DOR) of 62.0 (see Table 2). Posthoc thresholding based on rule‐out basis (high sensitivity), results in a sensitivity of 95.7% and specificity of 52.5% at the probability threshold of 0.223. On the other hand, posthoc thresholding based on rule‐in basis (high specificity), results in a sensitivity of 39.1% and specificity of 95.0% at the probability threshold of 0.875. The receiver operating curve is displayed in Figure 2, and the precision‐recall curve is shown in Figure 3. The calibration‐in‐the‐large of the model is −0.015, with a Brier‐score of 0.120 (cf. baseline prevalence 64% corresponding to a Brier‐score of 0.23). The calibration and decision curve are shown in Figure 4.

**TABLE 2:** Overview of the developed models and their performance metrics.

Metric	Full model	Baseline models		
		Volume	T2	FOS‐DCE
AUC	0.89 ± 0.06	0.51 ± 0.17	0.67 ± 0.10	0.82 ± 0.11
AUCPR	0.91 ± 0.06	0.70 ± 0.11	0.77 ± 0.09	0.88 ± 0.06
Threshold	0.625 ± 0.066	0.662 ± 0.036	0.623 ± 0.060	0.631 ± 0.012
Accuracy	89.0 ± 4.0%	45.9 ± 12.5%	68.0 ± 9.3%	81.4 ± 14.5%
Sensitivity	90.0 ± 6.6%	34.9 ± 19.6%	71.1 ± 19.4%	85.4 ± 7.6%
Specificity	87.7 ± 8.8%	65.0 ± 20.5%	62.5 ± 23.4%	75.0 ± 29.3%
DOR	62.0	1.0	4.1	17.7
Retained variance by PCA	98.8 ± 0.1%	n/a	90.6 ± 1.5%	81.8 ± 2.1%
Calibration‐in‐the‐large	−0.015	0.022	0.012	0.006
Brier score	0.12	0.24	0.22	0.15

*Note*: Data are either presented as mean ± standard deviation over all cross‐validation folds, or as a single overall figure.

Abbreviations: AUC, area under the receiver operating characteristic curve; AUCPR, area under the precision‐recall curve; DCE, dynamic contrast‐enhanced; DOR, diagnostic odds ratio; FOS, first‐order statistics; PCA, principal component analysis.

For the three baseline models, the best‐performing model based on solely tumor volume at diagnosis is an SVM with a linear kernel and yields a mean AUC of 0.51 ± 0.17 and AUCPR of 0.70 ± 0.11, with a mean accuracy of 45.9 ± 12.5%, sensitivity of 34.9 ± 19.6%, and specificity of 65.0 ± 20.5% at the optimal cut‐off probability of 0.662 ± 0.036. The calibration‐in‐the‐large of the model is 0.022, with a Brier score of 0.24. Second, the best‐performing T2‐based model is an SVM with a linear kernel (*N_bins_* = 32, *k* = 30, *n* = 5, variance retained by PCA = 90.6 ± 1.5%) and yields a mean AUC of 0.67 ± 0.10 and AUCPR of 0.77 ± 0.09, with a mean accuracy of 68.0 ± 9.3%, sensitivity of 71.1 ± 19.4%, and specificity of 62.5 ± 23.4% at the optical cut‐off probability of 0.623 ± 0.060. The calibration‐in‐the‐large of the model is 0.012, with a Brier score of 0.22. Third, the best‐performing model based on FOS of the DCE‐derived parameter maps is an SVM with a Gaussian kernel (*N_bins_* = 32, *k* = 20, *n* = 2, variance retained by PCA = 81.8 ± 2.1%) and yields a mean AUC of 0.82 ± 0.11 and AUCPR of 0.88 ± 0.06, with an overall accuracy of 81.4 ± 14.5%, sensitivity of 85.4 ± 7.6%, and specificity of 75.0 ± 29.3% at the optimal cut‐off probability of 0.631 ± 0.012. The calibration‐in‐the‐large of the model is 0.006, with a Brier score of 0.15. All receiver operating curves are displayed in Figure 2, and the precision‐recall curves are presented in Figure 3. Additionally, the calibration and decision curves are shown in Figure 4 and an overview of all performance metrics are summarize in Table 2.

The paired bootstrap test has demonstrated statistically significant differences between the overall AUC of all four models. The volume‐based model results in a mean difference of 0.114 (CI‐95%: −0.027–0.253, *P* = 0.002) compared to the T2‐based model, a mean difference of 0.318 (CI‐95%: 0.208–0.436, *P *< 0.001) compared to the FOS‐DCE model, and a mean difference of 0.370 (CI‐95%: 0.245–0.504, *P *< 0.001) compared to the main model. The T2‐based model yields a mean difference of 0.256 (CI‐95%: 0.121–0.382, *P* < 0.001) compared to the main model. Likewise, the model based on FOS of the DCE‐derived parameter maps shows a mean difference of 0.052 (CI‐95%: 0.008–0.103, *P* < 0.001) compared to the main model. Between the two baseline radiomic models, a mean difference of 0.204 (CI‐95%: 0.069– 0.341, *P* < 0.001) is observed.

### 3.3 Feature analysis

**FIGURE 5:**
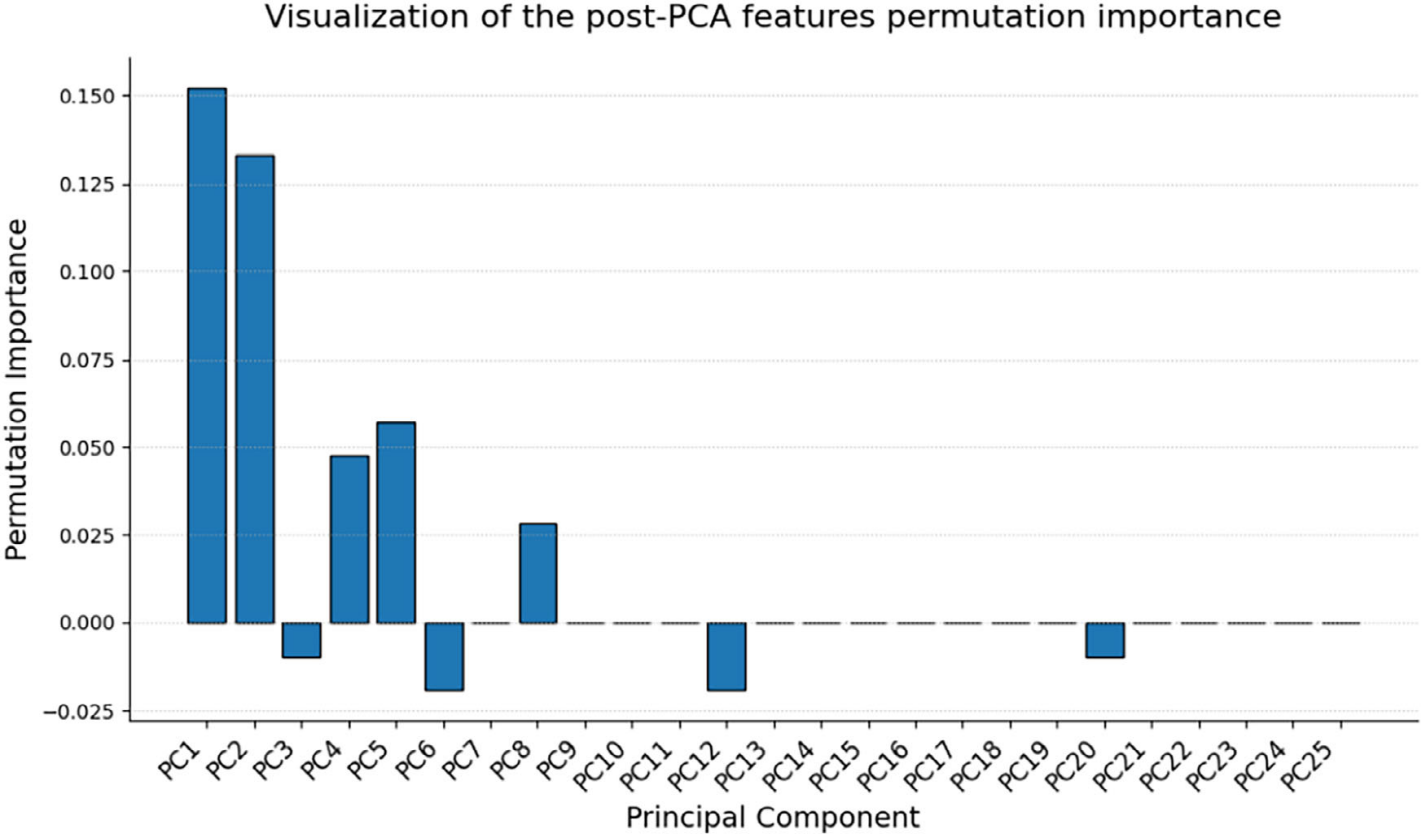
Bar chart showing the permutation importance of the resulting *n* = 25 principal components in the full model. Bar magnitude reflects the contribution of each component to the prediction of tumor growth. Negative values indicate a negative association, corresponding to tumor stability or regression. Principal components are ordered by explained variance.

The permutation importance of the *n *= 25 principal components is presented in Figure 5. The intersection of the resulting top‐*k* features of each of the five cross‐validation folds is calculated. This yields a set of 66 features that are exploited for model development in every single fold, which demonstrates a high degree of feature selection stability across folds. A total of 32 radiomic features derived from the K^trans^ parameter maps are selected, followed by 19 from the v_e_ parameter maps. The remaining 12 and 3 features are derived from the v_p_ parameter maps and T2‐weighted images, respectively.

The 66 selected features are grouped based on the representations that the features convey. This results in 9 feature groups, of which 3 correspond to K^trans^‐based features, another 3 to v_e_‐based features, 2 to v_p_‐based features, and 1 to T2‐based features. Table 3 summarizes the 9 grouped feature representations alongside their respective most discriminative feature type, based on the highest effect size, measured by Cohen's *d*. The mean and standard deviation of each corresponding feature type across all patients, stratified by growing and nongrowing tumors, are reported. The same data for all 66 selected features are reported in Table S‐1. For all features, a medium to large effect size is found between growing and nongrowing tumors.

**TABLE 3:** Overview of the identified feature representation groups with their corresponding most discriminative feature type for the prediction of vestibular schwannoma growth.

Modality	Feature representation group	Most discriminative feature type	Mean (standard deviation) feature value		Cohen's *d*
			Stable and regressing tumors	Growing tumors	
*K^trans^*	Value	(First order statistics) Mean	0.09 (0.06)	0.15 (0.08)	0.81
*K^trans^*	Heterogeneity	(GLSZM) Gray level non uniformity normalized	0.14 (0.07)	0.09 (0.05)	−0.91
*K^trans^*	Prominence of low gray values	(GLRLM) Short run low gray level emphasis	0.17 (0.11)	0.08 (0.07)	−0.99
*v_e_*	Variance	(First order statistics) Robust mean absolute deviation	0.12 (0.06)	0.07 (0.04)	−1.10
*v_e_*	Clustering	(GLCM) Cluster prominence	82 218 (67 535)	37 127 (46 922)	−1.00
*v_e_*	Heterogeneity	(GLCM) Difference average	7.3 (3.1)	4.8 (2.2)	−1.01
T2	Texture complexity	(GLCM) Correlation	0.68 (0.15)	0.76 (0.08)	0.72
*v_p_*	Value	(First order statistics) Mean	0.026 (0.016)	0.046 (0.031)	0.75
*v_p_*	Prominence of low gray values	(First order statistics) 10^th^ percentile	0.006 (0.009)	0.017 (0.014)	0.84

*Note*: The most discriminative feature type was identified based on the highest *t*‐statistic. *P*‐values correspond to the results of the independent *t*‐test comparing growing and nongrowing tumors for the most discriminative feature type.

Abbreviations: GLSZM, gray‐level size‐zone matrix; GLRLM, gray‐level run‐length matrix; GLCM, gray‐level co‐occurrence matrix.

**FIGURE 6:**
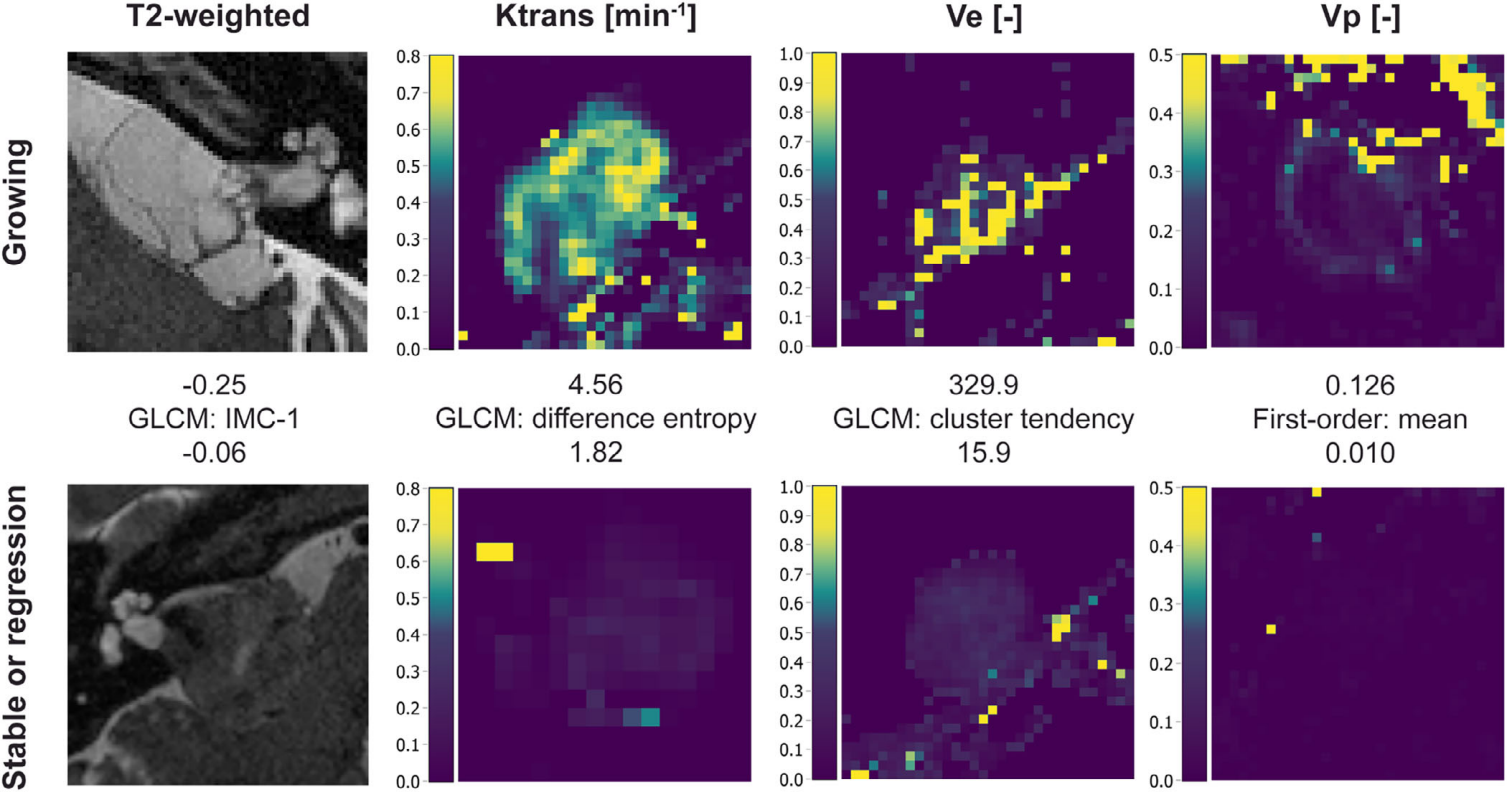
Case examples of structural T2‐weighted imaging and the DCE‐derived parameter maps (i.e. K^trans^, v_e_, and v_p_) for growing and nongrowing (stable or regressing) tumors with the corresponding measured illustrative feature value. GLCM, gray‐level cooccurrence matrix; IMC, informational measure of correlation.

According to this feature analysis, tumor growth corresponds to higher K^trans^ and v_p_ parameter values, heterogeneity of parameter values in K^trans^, and more complex textures in T2‐weighted images. Conversely, tumor stability or regression is associated with a high variance and heterogeneity in v_e_ parameter values and the respective spatial clustering of similar values. Examples of the structural imaging and each DCE‐derived parameter map with corresponding illustrative feature values for both growing and nongrowing tumors with are depicted in Figure 6.

## 4 DISCUSSION

Personalized clinical decision‐making for VS patients assigned to a W&S strategy will be greatly improved if tumor growth can be predicted at or soon after diagnosis.^11^ Even though VS are generally considered to be unpredictable in nature,^2^ several studies that either employ dynamic MRI or AI‐based models have demonstrated promising results.^12^, ^14^, ^16^, ^23^, ^24^, ^25^, ^26^ Therefore, this study has investigated the use of machine learning, employing both structural and dynamic MRI, for the prediction of short‐term growth in untreated sporadic VS.

### 4.1 Evaluation of model performance

The developed SVM prediction model, based on radiomic features of T2‐weighted scans and DCE‐derived parameter maps, yields an excellent internally cross‐validated performance with a mean AUC of 0.89 ± 0.06. Additionally, an outstanding mean accuracy of 89.0%, sensitivity of 90.0%, and specificity of 87.7% are found at the optimal cut‐off probability of 0.481. Furthermore, the model shows an FPR of 12.3% and FNR of 10.0%, indicating that the model is more tuned toward overpredicting tumor growth. This increased sensitivity, which is often desirable in clinical practice,^32^ can be attributed to the overrepresentation of growing tumors in the dataset. However, the degree of overprediction is very low, as the DOR is measured at 62.0. This indicates that the odds of accurately predicting tumor growth are 62.0 times higher than the odds of falsely predicting a nongrowing tumor to be growing (i.e. a false positive). Furthermore, the model is well calibrated, which is reflected by an almost diagonal calibration curve, near‐zero calibration‐in‐the‐large and a Brier score of 0.12.^33^ Additionally, the decision curve analysis indicates that the model provides a higher net benefit than both treat‐all and treat‐none strategies across threshold probabilities from 0.0 to 0.9. However, for extremely high thresholds, the net benefit falls below the threat‐none strategy, reflecting limited utility in this range.^34^

The full model considerably outperforms the baseline models and existing growth prediction models for untreated sporadic VS in terms of discrimination and calibration. These already conducted studies employed either less complex statistical models,^14^ or relied on solely clinical variables (e.g. patient age, tumor cisternal extent, macrocystic change, and prior tumor growth) and/or structural imaging as input data.^12^, ^16^, ^35^ Therefore, the obtained improvement in model performance may be attributed to two key factors. First, the use of machine learning methods, of which the capabilities are further exemplified by the performance difference with the logistic regression model that employed the same dataset, as developed by Schouten et al. (cf. AUC of 0.85 and DOR of 21.9).^14^ Second, the highly predictive value of DCE‐derived parameters in comparison to structural imaging. This high prediction value is reflected by both the lower performance of the T2‐based baseline model compared to the models employing DCE‐based features, and the limited amount of structural imaging‐based features employed in each cross‐validation fold compared to the amount of DCE‐based features.

### 4.2 Predictive value of radiomic features

Overall, the K^trans^ and v_e_ parameter maps contribute most of the features to the developed prediction model. Their important predictive value has been described in earlier studies.^14^, ^17^ Consistent with these studies, higher K^trans^ values throughout the tumor are associated with progression. For v_e_ values, more complex radiomic features are found more discriminative than mean tumor values. Instead of an inverse correlation between mean *v_e_* values and tumor growth,^14^ homogeneity and a lower variance of v_e_ values are considered indicative of tumor growth. Conversely, the tendency of similar v_e_ values to cluster together within the tumor is associated with tumor stability or regression. This indicates that even though v_e_ values in nongrowing tumors vary greatly throughout the tumor, they exhibit spatial coherence, where neighboring voxels have similar values. Additionally, heterogeneity of K^tran^ *^s^* values inside the tumor is associated with tumor stability or regression. This added predictive value of more complex radiomic features is demonstrated by the statistically significant performance difference between the baseline model employing solely FOS of the DCE‐derived parameter maps and the model using the complete feature set. This finding has been described in earlier studies as well,^24^, ^36^ further demonstrating their high predictive potential.

The model utilizes the least amount of features from the v_p_ parameter maps and structural T2‐weighted imaging data. In the small pilot study by Lewis et al.,^17^ a no‐significant trend to higher v_p_ values in growing tumors was measured. In the much larger study by Schouten et al.,^14^ v_p_ has demonstrated a significant association with tumor growth on univariate analyses, but only the fourth quartile of mean v_p_ values of >0.06 has exhibited a significant OR of 3.9 for growth. Given the previously demonstrated association between tumor CD31^+^ microvessel density (as measured on histological analyses) and measures of both tumor K^trans^ and v_p_, a coincrease in both of these parameters in growing VS is expected.^17^, ^19^ Indeed, the more robust association between tumor v_p_ values and growth shown in this study may reflect not only differences in scanner acquisition and increased sample size compared to earlier smaller studies, but also importantly refer to the added predictive value of the more complex radiomic features used.^24^, ^36^, ^37^

A final factor demonstrated in this study to be associated with tumor growth is tumor texture complexity in T2‐weighted imaging. A study employing T1‐weighted imaging has reported tumor heterogeneity, closely related to complexity, to be predictive of tumor growth.^16^ Moreover, texture heterogeneity has been associated with hearing loss,^38^, ^39^ and although conflicting reports exist,^13^ hearing loss has been linked to tumor growth in some studies.^40^

### 4.3 Association between radiomics and local inflammation

Possible associations between imaging‐based biomarkers and VS tumor histology or microenvironmental properties have been investigated extensively in literature.^17^, ^19^, ^20^, ^36^, ^41^ Texture complexity in structural imaging has been linked to tumor histology and specifically, the presence of inflammatory cells,^36^ which in turn has been associated with tumor growth and local inflammation.^42^, ^43^ DCE‐MRI derived K^trans^ in particular has been considered to be a potential indirect measure of inflammatory response in VS, since it scales with tumor‐associated macrophage (TAM) infiltration.^14^, ^19^ The developed model attaches considerable importance to K^tran^ *^s^*‐based features and tumor texture complexity for the prediction of VS tumor growth. Since both of these feature types have been linked to tumor growth and local tumor inflammation, further evidence is provided that tumor growth in untreated VS may be related to local inflammatory processes. Despite the increasing evidence of this association, the etiology of the inflammation remains unknown. To further investigate this hypothesis, future work should not only focus on the relationship between radiomics and specific immunohistological markers, but also investigate the etiology related to the tumor microenvironment.

### 4.4 Limitations

This study is constrained by three limitations, addressing model generalization, stability of measurement results, and model consistency with existing literature.

First, the generalization of the developed model to other centers is currently unknown. The imaging data was acquired in a single center, using a single type of MRI scanner with an identical acquisition protocol across the study population. However, some evidence exists that DCE‐parameter values in VS may be fairly consistent and robust across different scanners, field strengths, and protocols, particularly for highly vascular tumors.^14^, ^17^ This is exemplified by the similarity between the parameter value distributions found in this cohort^14^ and those reported in an earlier study employing a different scanner and protocol.^17^ This increases the likelihood of good model generalization of the method. It should be noted that the developed model employs T2‐weighted images as well, which are generally considered to be highly heterogeneous across different scanners.^44^ However, a previous study has shown that models based on radiomics of single‐center T2‐weighted images may exhibit limited generalization.^45^ Therefore, the generalization of the proposed model and thereby the reproducibility of this study should be investigated in future work, by externally validating the radiomic feature values and the performance of the current model. Finally, an updated model should be developed using multicenter data.

Second, studies provide evidence that the identified key radiomic features exhibit high test‐retest stability, with intraclass correlation coefficients exceeding 0.8 for T2‐weighted images.^46^, ^47^ Although some studies have demonstrated a high consistency of DCE biomarkers as well,^48^ the stability of DCE‐derived radiomics remains poorly understood. Future work should therefore thoroughly evaluate the reproducibility and consistency of the corresponding radiomic features across repeated measurements.

Lastly, the model is developed using a dimensionality‐reduced feature vector, which means that the model employs a linear combination of radiomic features for its prediction. Although the permutation importance analysis provides some insight into the importance of the principal components, interpretation remains challenging due to the loss of direct correspondence to meaningful features and the nonlinearity of the model. Therefore, the feature analysis is based on the features prior to dimensionality reduction. Only indirect associations can therefore be made between the analyzed feature types and their respective discriminative ability for the model. Since the found predictive feature types in this study are consistent with existing literature, there are limited grounds to dispute the predictive value of the found feature types.

## 5 CONCLUSION

The developed model demonstrates the high potential of the combination of machine learning techniques and dynamic MRI for the prediction of tumor growth in VS patients. The DCE‐derived parameters show high predictive value and provide insight into possible links between tumor biology and growth mechanisms. Furthermore, radiomic features have revealed to be superior to FOS as predictors for VS tumor growth, while preserving a degree of model explainability. Nevertheless, before the model can be implemented into clinical practice, the reproducibility of the radiomics and model itself should be investigated.

## CONFLICT OF INTEREST STATEMENT

Patrick P.J.H. Langenhuizen is partly funded by Elekta AB (Stockholm, Sweden).

## Supporting information



Supporting Information

Supporting Information
