# Supplementary material for: Computer‐aided prediction of growth in vestibular schwannomas based on both structural and dynamic contrast‐enhanced MR imaging
Source: Med Phys. 2025 Dec 29;53(1):e70224. doi: 10.1002/mp.70224 (PMC12746057; doi:10.1002/mp.70224)
Supplement: Supplementary file 1 — Supporting Information [file MP-53-0-s002.doc]

# Supplementary methods

## MRI acquisition protocol

The employed MRI acquisition protocol is identical to our prior study, as it employs a subset of the previously reported dataset^1^.

MRI examinations were performed using a 3T MAGNETOM Prismafit scanner (Siemens Healthineers, Erlangen, Germany) running software version VE11C, and a dedicated 20-channel head and neck phased-array coil. Images were obtained in axial orientation using the following sequences:

(1) 3D turbo spin-echo (SPACE) T2-weighted imaging with TR/TE of 1000/139 msec, refocusing flip angle of 120°, field of view (FOV) of 200 × 200 mm^2^, matrix size of 384 × 384, voxel size of 0.5 × 0.5 × 0.5 mm (interpolated from 0.5 × 0.5 × 1.0 mm), and acquisition time of 4 min 18 sec;

(2) Pre-contrast quantitative T1 mapping using a series of 3D spoiled gradient echo (GRE) acquisitions with flip angles of 2°, 6°, 12° and 16°, TR/TE of 9.0/4.14 msec, FOV of 192 × 192 mm^2^, matrix size of 160 × 160, voxel size of 1.2 × 1.2 × 2.5 mm (interpolated from 1.2 × 1.3 × 3.2 mm), acceleration factor 2, and acquisition time of 1 min 52 sec;

(3) Dynamic contrast-enhanced T1-weighted imaging using a Time-resolved angiography With Interleaved Stochastic Trajectories (TWIST) sequence with TR/TE of 3.2/1.1 msec, flip angle of 14°, the same FOV, matrix and voxel size as the T1 mapping, acceleration factor 2, temporal resolution of 2.35 sec, and an a total acquisition time of 5 min 23 sec. After obtainment of five non-contrast baseline frames a single intravenous bolus injection of 0.1 mmol/kg gadoteric acid (Dotarem, Guerbet, Villepinte, France) was administered with an injection rate of 2.5 ml/s followed by a 20 ml saline flush.

## 2. Dynamic contrast-enhanced MRI

The employed acquisition and analysis protocol for the dynamic contrast-enhanced MRI is identical to our prior study, as it employs a subset of the previously reported dataset^1^.

### Acquisition

Data were acquired using a single injection dynamic contrast enhanced MRI technique. Pre-contrast quantitative T1 mapping was performed using a series of 3D spoiled gradient echo (GRE) acquisitions with flip angles of 2°, 6°, 12° and 16°, TR/TE of 9.0/4.14 msec, FOV of 192 × 192 mm^2^, matrix size of 160 × 160, voxel size of 1.2 × 1.2 × 2.5 mm (interpolated from 1.2 × 1.3 × 3.2 mm), acceleration factor 2, and acquisition time of 1 min 52 sec. Dynamic contrast-enhanced T1-weighted imaging was then acquired using a Time-resolved angiography With Interleaved Stochastic Trajectories (TWIST) sequence with TR/TE of 3.2/1.1 msec, flip angle of 14°, the same FOV, matrix and voxel size as the T1 mapping, acceleration factor 2, temporal resolution of 2.35 sec, and a total acquisition time of 5 min 23 sec. During image acquisition and after obtainment of five non-contrast baseline frames an intravenous bolus injection of 0.1 mmol/kg gadoteric acid (Dotarem, Guerbet, Villepinte, France) was administered followed by a 20 ml saline flush.

### Analysis

Modelling of DCE-MRI derived kinetic parameters requires identification of a suitable vascular input function (VIF). An experimentally derived, population-averaged fixed VIF can be used for all subjects^2–4^, but large variations in the actual VIF can occur between subjects due to physiological (e.g. haematocrit, caffeine intake, cardiac output, and atherosclerosis related vessel narrowing) and technical factors (e.g. differences in dose and injection timing)^2,5,6^. For defining an individualised VIF, the feeding artery of the tumor is the ideal choice but is often not possible due to the small size of the feeding vessel or its absence within the imaging field of view (FOV)^2,6–9^. Large intracranial arteries such as the internal carotid artery (ICA) and middle cerebral artery (MCA), and even large venous drainage channels such as the superior sagittal sinus (SSS) are therefore often used to provide surrogate global VIF measurements for each individual^6^. For this study, plasma GBCA concentration-time curves, C_p_(t), were derived from signal intensity (SI)—time curves measured in the horizontal segment of the middle cerebral artery (MCA) for each patient. In particular we elected to use a VIF extracted from the MCA for this study, as firstly unlike input estimation from the SSS it does not require a large volume, whole brain DCE-MRI acquisition to reduce blood inflow-induced errors; and secondly because in an earlier published study we demonstrated that compared to other large arteries such as the internal carotid artery the MCA provides a superior VIF with higher peak and greater sensitivity to interindividual changes in plasma GBCA concentration^6^.

Prior to kinetic fitting and for measurement of VIF in the MCA, a pair of rectangle ROIs was manually drawn on the horizontal segment of the MCA bilaterally, and an automatic method was then used to search and identify voxels within neighbouring contiguous axial slices that displayed maximum enhancement area under the SI curve within 30 seconds of the bolus arrival time (AUC_30_)^10,11^. A mean SI-time curve was then calculated from 20 voxels with the highest AUC_30_. This mean SI-time curve was then converted to a plasma GBCA concentration-time curve C_p_(t) using previously described methods^12^. For the conversion to a concentration-time curve a literature value of blood R1_0_ of 0.694 s^-1^ was used, due to the difficulties of accurately measuring the pre-contrast T1 of flowing blood using standard DCE-MRI sequences and the bias introduced through in vivo experiments^13–16^.

For each tumor and brain tissue voxel the SI-time curves were converted to tissue GBCA concentration curves using measured voxelwise values of tissue native longitudinal relaxation rate (R1_N_) derived from the pre-contrast quantitative T1 mapping series_._ The voxelwise tissue GBCA concentration-time curves from each patient were then fitted to the extended Tofts model (ETM), resulting in the found K_trans_, v_e_, and v_p_ values. As part of this procedure, and prior to kinetic fitting, the bolus arrival time (BAT) for each tissue voxel was estimated and the AIF (C_p_(t)) measured from the MCA time-shifted to align with the BAT of each tissue GBCA concentration-time curve.

ETM fitting was performed using a simplex annealing optimisation algorithm^17,18^, which is a hybrid global-to-local search technique that combines the Nelder–Mead simplex method^19–21^ with simulated annealing^20–24^. This allows exploration of different parameter values broadly at first before converging precisely to a minimum, and allows for both fast local convergence due to the simplex method and the ability to escape local minima due to annealing^17^. Initial values for K^trans,^ v_e_, and v_p_ were set based on their theoretical physiologic ranges, and appropriate parameter bounds were applied. Convergence criteria and failed-fit handling were also defined within the optimisation routine

To assess the discrepancy between the derived curve and the original data map of scaled fitting error (SFE)^25,26^ was generated using the equation below:

$$SFE = \surd\left( \frac{\left( \Sigma\left( T_{i}- A_{i} \right)^{2} \right)}{\Sigma A_{i}^{2}} \right)\times100\%$$

Where Ti and Ai are the theoretical and experimental gadolinium concentration values at each dynamic time-point, respectively and with each residual reflecting the difference between the measured concentration value and the predicted value from the ETM fit. Tumor voxels with an SFE value above 50% were excluded from the statistics and in all cases to confirm the acceptance of the use of SFE > 50% for excluding outlier tumor voxels, visual inspection of derived SFE and kinetic parameter maps (before and after exclusion of voxels with SFE > 50%) was performed. Prior DCE-MRI studies in patients with CNS tumours have demonstrated that the majority of enhancing voxels within the tumor volume had a SFE < 30%^27,28^,with surrounding non-enhancing tissue voxels usually displaying an SFE > 80%. An SFE threshold value of 50% was therefore empirically determined to be the optimum threshold for removing poorly fitting, noisy voxels whilst maintaining the majority of enhancing tumour^29^. Furthermore, prior DCE-MRI studies in VS specifically have demonstrated that use of this 50% threshold gives kinetic parameters that: correlate strongly with tissue markers of tumour vascularity and the extravascular-extracellular space (EES)^26,30,31^; have predictive power in determining the treatment response of NF2-schwannommatosis related VS to bevacizumab treatment^32,33^; and predictive ability for future tumor growth in large VS cohorts^1^.

### Sensitivity analysis of the SFE

The exclusion criterion for voxels with SFE > 50% resulted in a median per-patient exclusion rate of 0.0%, mean of 2.6%, and standard deviation of 6.2%. For a total of 59 patients (53.6%) no voxels were excluded, as all voxels had an SFE < 50%.

A brief sensitivity analysis was conducted to assess the effect of the SFE exclusion threshold on the mean percentage of excluded voxels (see figure below). There is a steep drop in exclusion percentage for the thresholds between 0% and 20%, which then gradually flattens at higher threshold values. This indicates that most voxels within the VS tumor have low SFE values. Consequently, the sensitivity gain obtainable with higher threshold values is marginal. Additionally, an SFE exclusion threshold of 50% was found to yield maps with the best tissue correlation^6^ and predictive ability for tumor growth^1^, further reinforcing the choice of a 50% threshold.


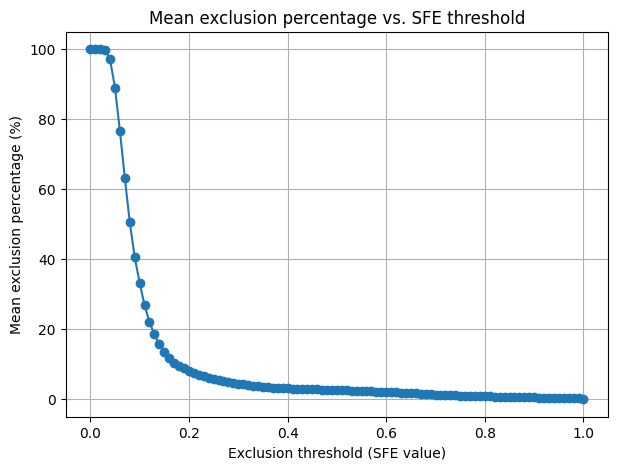


## 3. Segmentation variability due to morphological operations

Due to the lower spatial resolution of DCE imaging sequences and potentially resulting partial volume effects, morphological erosion was applied to the tumor annotations. In order to assess the effects of morphological operations (i.e. erosion and its counterpart, dilation) on the original annotations, the Dice-score and volume difference are calculated (see table below). A 3x3 cross-shaped structural contour filter is employed. The resulting Dice-score for a single morphological operation are high (i.e. 0.88 and 0.90 for erosion and dilation, respectively), reflecting a strong similarity to the original annotation. This suggests that the information loss due to a single erosion operation is minimal, while ensuring that all voxels inside the ROI are fully captured within the tumor contour.

| Operation | Dice-score [-] | Volume difference [mm^3^] |
| --- | --- | --- |
| Erosion (2-times) | 0.75 (0.64 – 0.84) | -330 (-704 – -136) |
| **Erosion (1-time)** | 0.88 (0.83 – 0.92) | -176 (-366 – -73) |
| Original (reference) | n/a | n/a |
| Dilation (1-time) | 0.90 (0.86 – 0.93) | 190 (85 – 385) |
| Dilation (2-times) | 0.81 (0.75 – 0.87) | 393 (171 – 788) |
| **Note.** Data is presented as median with inter-quartile range in parentheses. The erosion (1-time) operation is employed in this study. | | |

## 4. Final PyRadiomics configuration

| Setting | Configuration |
| --- | --- |
| **Version** | 3.1.0 |
| **Used image type** | Original (no filter applied) |
| **Image discretization** | Fixed-bin width |
| - **Bin width** | [mean voxel intensity of modality] / N_bin |
| - - K_trans | 0.581 / N_bin [1/min] |
| - - v_e | 0.874 / N_bin [-] |
| - - v_p | 0.177 / N_bin [-] |
| - - T2 | 5.174 / N_bin [-] |
| - **N_bin** | 32 (tunable hyper-parameter) |
| **Enabled features** | Model dependent (all; only T2; only DCE first-order radiomics) |
| **Image normalization** | False (Note: image normalization for T2 is executed in a separate part of our code) |
| **Resampling** | None |
| **Other settings** | Default value per version 3.1.0 |

### References

1. Schouten SM, Lewis D, Cornelissen S, et al. Dynamic contrast-enhanced and diffusion-weighted MR imaging for predicting tumor growth of sporadic vestibular schwannomas: a prospective study. *Neuro Oncol*. Published online November 23, 2024. doi:10.1093/neuonc/noae252

2. Yankeelov T, Gore J. Dynamic Contrast Enhanced Magnetic Resonance Imaging in Oncology:Theory, Data Acquisition,Analysis, and Examples. *Curr Med Imaging Rev*. 2007;3(2):91-107. doi:10.2174/157340507780619179

3. Weinmann HJ, Laniado M, Mützel W. Pharmacokinetics of GdDTPA/dimeglumine after intravenous injection into healthy  volunteers. *Physiol Chem Phys Med NMR*. 1984;16(2):167-172.

4. Parker GJM, Roberts C, Macdonald A, et al. Experimentally-derived functional form for a population-averaged  high-temporal-resolution arterial input function for dynamic contrast-enhanced MRI. *Magn Reson Med*. 2006;56(5):993-1000. doi:10.1002/mrm.21066

5. Buckley DL. Uncertainty in the analysis of tracer kinetics using dynamic contrast-enhanced T1-weighted MRI. *Magn Reson Med*. 2002;47(3):601-606. doi:10.1002/mrm.10080

6. Lewis D, Zhu X, Coope DJ, et al. Surrogate vascular input function measurements from the superior sagittal sinus  are repeatable and provide tissue-validated kinetic parameters in brain DCE-MRI. *Sci Rep*. 2022;12(1):8737. doi:10.1038/s41598-022-12582-x

7. Li KL, Lewis D, Jackson A, Zhao S, Zhu X. Low-dose T1W DCE-MRI for early time points perfusion measurement in patients with intracranial tumors: A pilot study applying the microsphere model to measure absolute cerebral blood flow. *Journal of Magnetic Resonance Imaging*. 2018;48(2):543-557. doi:10.1002/jmri.25979

8. Yang C, Stadler WM, Karczmar GS, Milosevic M, Yeung I, Haider MA. Comparison of quantitative parameters in cervix cancer measured by dynamic  contrast-enhanced MRI and CT. *Magn Reson Med*. 2010;63(6):1601-1609. doi:10.1002/mrm.22371

9. Sourbron S, Ingrisch M, Siefert A, Reiser M, Herrmann K. Quantification of cerebral blood flow, cerebral blood volume, and blood-brain-barrier leakage with DCE-MRI. *Magn Reson Med*. 2009;62(1):205-217. doi:10.1002/mrm.22005

10. Li KL, Zhu X, Zhao S, Jackson A. Blood–brain barrier permeability of normal-appearing white matter in patients with vestibular schwannoma: A new hybrid approach for analysis of T<inf>1</inf>-W DCE-MRI. *Journal of Magnetic Resonance Imaging*. Published online 2017. doi:10.1002/jmri.25573

11. Li KL, Lewis D, Coope DJ, et al. The LEGATOS technique: A new tissue-validated dynamic contrast-enhanced MRI method  for whole-brain, high-spatial resolution parametric mapping. *Magn Reson Med*. Published online May 2021. doi:10.1002/mrm.28842

12. Zhu XP, Li KL, Kamaly-Asl ID, et al. Quantification of endothelial permeability, leakage space, and blood volume in brain tumors using combined T1 and T2* contrast-enhanced dynamic MR imaging. *Journal of Magnetic Resonance Imaging*. 2000;11(6):575-585. doi:10.1002/1522-2586(200006)11:6<575::AID-JMRI2>3.0.CO;2-1

13. Bourassa-Moreau B, Lebel R, Gilbert G, Mathieu D, Lepage M. Robust arterial input function surrogate measurement from the superior sagittal  sinus complex signal for fast dynamic contrast-enhanced MRI in the brain. *Magn Reson Med*. Published online July 2021. doi:10.1002/mrm.28922

14. Duan C, Kallehauge JF, Bretthorst GL, Tanderup K, Ackerman JJH, Garbow JR. Are complex DCE-MRI models supported by clinical data? *Magn Reson Med*. 2017;77(3):1329-1339. doi:10.1002/mrm.26189

15. Stanisz GJ, Odrobina EE, Pun J, et al. T1, T2 relaxation and magnetization transfer in tissue at 3T. *Magn Reson Med*. 2005;54(3):507-512. doi:10.1002/mrm.20605

16. Shen Y, Goerner FL, Snyder C, et al. T1 relaxivities of gadolinium-based magnetic resonance contrast agents in human  whole blood at 1.5, 3, and 7 T. *Invest Radiol*. 2015;50(5):330-338. doi:10.1097/RLI.0000000000000132

17. G. W, Vetterling WT, Teukolsky SA, Press WH, Flannery BP. Numerical Recipes Example Book (C). *Math Comput*. 1989;52(185):253. doi:10.2307/2008674

18. Ingber L. Simulated annealing: Practice versus theory. *Math Comput Model*. 1993;18(11):29-57. doi:10.1016/0895-7177(93)90204-C

19. Nelder JA, Mead R. A Simplex Method for Function Minimization. *Comput J*. 1965;7(4):308-313. doi:10.1093/comjnl/7.4.308

20. Svistoun I, Driscoll B, Coolens C. Accuracy and performance of functional parameter estimation using a novel numerical optimization approach for gpu-based kinetic compartmental modeling. *Tomography*. 2019;5(1):209-219. doi:10.18383/j.tom.2018.00048

21. Hsu YHH, Huang Z, Ferl GZ, Ng CM. GPU-accelerated compartmental modeling analysis of DCE-MRI data from glioblastoma patients treated with bevacizumab. *PLoS One*. 2015;10(3):e0118421. doi:10.1371/journal.pone.0118421

22. Kirkpatrick S, Gelatt CD, Vecchi MP. Optimization by simulated annealing. *Science (1979)*. 1983;220(4598):671-680. doi:10.1126/science.220.4598.671

23. El Amoury S, Smili Y, Fakhri Y. Simulated Annealing-Based Hyperparameter Optimization of a Convolutional Neural Network for MRI Brain Tumor Classification. *Mach Learn Knowl Extr*. 2025;7(2):50. doi:10.3390/make7020050

24. Li X, Zhang P, Brisman R, Kutcher G. Use of simulated annealing for optimization of alignment parameters in limited MRI acquisition volumes of the brain. *Med Phys*. 2005;32(7):2363-2370. doi:10.1118/1.1944287

25. Li KL, Wilmes LJ, Henry RG, et al. Heterogeneity in the angiogenic response of a BT474 human breast cancer to a novel vascular endothelial growth factor-receptor tyrosine kinase inhibitor: Assessment by voxel analysis of dynamic contrast-enhanced MRI. *Journal of Magnetic Resonance Imaging*. 2005;22(4):511-519. doi:10.1002/jmri.20387

26. Li KL, Lewis D, Coope DJ, et al. The LEGATOS technique: A new tissue-validated dynamic contrast-enhanced MRI method for whole-brain, high-spatial resolution parametric mapping. *Magn Reson Med*. 2021;86(4):2122-2136. doi:10.1002/mrm.28842

27. Zhu XP, Li KL, Kamaly-Asl ID, et al. Quantification of endothelial permeability, leakage space, and blood volume in brain tumors using combined T1 and T2* contrast-enhanced dynamic MR imaging. *Journal of Magnetic Resonance Imaging*. 2000;11(6):575-585. doi:10.1002/1522-2586(200006)11:6<575::AID-JMRI2>3.0.CO;2-1

28. Lewis D, Li KL, Waqar M, et al. Low-dose GBCA administration for brain tumour dynamic contrast enhanced MRI: a feasibility study. *Sci Rep*. 2024;14(1):4905. doi:10.1038/s41598-024-53871-x

29. Li KL, Buonaccorsi G, Thompson G, et al. An improved coverage and spatial resolutiona-using dual injection dynamic contrast-enhanced (ICE-DICE) MRI: A novel dynamic contrast-enhanced technique for cerebral tumors. *Magn Reson Med*. 2012;68(2):452-462. doi:10.1002/mrm.23252

30. Lewis D, Roncaroli F, Agushi E, et al. Inflammation and vascular permeability correlate with growth in sporadic vestibular schwannoma. *Neuro Oncol*. 2019;21(3):314-325. doi:10.1093/neuonc/noy177

31. Lewis D, Donofrio CA, O’Leary C, et al. The microenvironment in sporadic and neurofibromatosis type II–related vestibular schwannoma: The same tumor or different? A comparative imaging and neuropathology study. *J Neurosurg*. 2021;134(5):1419-1429. doi:10.3171/2020.3.JNS193230

32. Li KL, Djoukhadar I, Zhu X, et al. Vascular biomarkers derived from dynamic contrast-enhanced MRI predict response of vestibular schwannoma to antiangiogenic therapy in type 2 neurofibromatosis. *Neuro Oncol*. 2016;18(2):275-282. doi:10.1093/neuonc/nov168

33. Li KL, Lewis D, Zhu X, et al. A Novel Multi-Model High Spatial Resolution Method for Analysis of DCE MRI Data: Insights from Vestibular Schwannoma Responses to Antiangiogenic Therapy in Type II Neurofibromatosis. *Pharmaceuticals*. 2023;16(9):1282. doi:10.3390/ph16091282
