# Supplementary material for: Computer‐aided prediction of growth in vestibular schwannomas based on both structural and dynamic contrast‐enhanced MR imaging
Source: Med Phys. 2025 Dec 29;53(1):e70224. doi: 10.1002/mp.70224 (PMC12746057; doi:10.1002/mp.70224)
Supplement: Supplementary file 2 — Supporting Information [file MP-53-0-s001.docx]

| Supplementary Table 1. Statistics of all 66 selected features | | | | | | |  |
| --- | --- | --- | --- | --- | --- | --- | --- |
| Modality | **Feature representation group** | **Feature category** | **Feature type** | **Mean feature value with corresponding standard deviation** | | **Cohen’s-*d* (CI-95%)** | |
|  |  |  |  | Stable and regressing tumors | Growing tumors |  |  |
| *K^trans^* | Value | First order statistics | 90^th^ percentile | 0.14 ± 0.09 | 0.24 ± 0.15 | 0.72 (0.32, 1.13 | |
| *K^trans^* | Value | First order statistics | Mean | 0.09 ± 0.06 | 0.15 ± 0.08 | 0.81 (0.40, 1.21) | |
| *K^trans^* | Value | First order statistics | Median | 0.08 ± 0.05 | 0.13 ± 0.07 | 0.76 (0.36, 1.16) | |
| *K^trans^* | Value | First order statistics | Root mean square | 0.10 ± 0.06 | 0.17 ± 0.11 | 0.80 (0.39, 1.20) | |
| *K^trans^* | Value | GLCM | Joint average | 4.96 ± 2.97 | 7.74 ± 4.40 | 0.71 (0.31, 1.11) | |
| *K^trans^* | Value | GLDM | Large dependence high gray level emphasis | 447.6 ± 671.2 | 235.5 ± 434.8 | 0.59 (0.19, 0.99) | |
| *K^trans^* | Value | GLSZM | High gray level zone emphasis | 59.9 ± 66.3 | 168.7 ± 227.5 | 0.58 (0.18, 0.98) | |
| *K^trans^* | Value | GLSZM | Small area high gray level emphasis | 45.8 ± 58.0 | 144.7 ± 216.4 | 0.56 (0.16, 0.95) | |
|  | | | | | | | |
| *K^trans^* | Heterogeneity | First order statistics | Entropy | 2.81 ± 0.79 | 3.43 ± 0.80 | 0.78 (0.38, 1.18) | |
| *K^trans^* | Heterogeneity | First order statistics | Mean absolute deviation | 0.04 ± 0.02 | 0.06 ± 0.04 | 0.60 (0.20, 1.00) | |
| *K^trans^* | Heterogeneity | First order statistics | Uniformity | 0.20 ± 0.12 | 0.13 ± 0.07 | -0.71 (-1.11, -0.31) | |
| *K^trans^* | Heterogeneity | GLCM | Difference entropy | 2.27 ± 0.67 | 2.77 ± 0.73 | 0.71 (0.31, 1.11) | |
| *K^trans^* | Heterogeneity | GLCM | Id | 0.51 ± 0.14 | 0.41 ± 0.12 | -0.75 (-1.15, -0.35) | |
| *K^trans^* | Heterogeneity | GLCM | Idm | 0.45 ± 0.16 | 0.34 ± 0.14 | -0.74 (-1.15, -0.35) | |
| *K^trans^* | Heterogeneity | GLCM | Maximum probability | 0.14 ± 0.13 | 0.07 ± 0.06 | -0.66 (-1.06, -0.26) | |
| *K^trans^* | Heterogeneity | GLCM | Sum average | 9.91 ± 5.93 | 15.47 ± 8.79 | 0.71 (0.31, 1.11) | |
| *K^trans^* | Heterogeneity | GLDM | Small dependence emphasis | 0.22 ± 0.14 | 0.32 ± 0.17 | 0.65 (0.25, 1.05) | |
| *K^trans^* | Heterogeneity | GLRLM | Gray level non uniformity normalized | 0.18 ± 0.10 | 0.12 ± 0.07 | -0.71 (-1.11, -0.31) | |
| *K^trans^* | Heterogeneity | GLRLM | Run entropy | 3.40 ± 0.65 | 3.87 ± 0.65 | 0.73 (0.33, 1.13) | |
| *K^trans^* | Heterogeneity | GLRLM | Run length non uniformity normalized | 0.76 ± 0.11 | 0.83 ± 0.10 | 0.69 (0.29, 1.09) | |
| *K^trans^* | Heterogeneity | GLRLM | Run percentage | 0.86 ± 0.07 | 0.90 ± 0.06 | 0.66 (0.26, 1.06) | |
| *K^trans^* | Heterogeneity | GLRLM | Short run emphasis | 0.89 ± 0.06 | 0.92 ± 0.05 | 0.69 (0.29, 1.09) | |
| *K^trans^* | Heterogeneity | GLSZM | Gray level non uniformity normalized | 0.14 ± 0.07 | 0.09 ± 0.05 | -0.91 (-1.32, -0.50) | |
| *K^trans^* | Heterogeneity | GLSZM | Small area emphasis | 0.55 ± 0.12 | 0.64 ± 0.10 | 0.83 (0.43, 1.24) | |
| *K^trans^* | Heterogeneity | GLSZM | Zone entropy | 4.17 ± 1.12 | 4.91 ± 0.89 | 0.75 (0.35, 1.16) | |
| *K^trans^* | Heterogeneity | GLSZM | Zone percentage | 0.26 ± 0.18 | 0.38 ± 0.20 | 0.61 (0.22, 1.01) | |
|  |  | | | | | | |
| *K^trans^* | Prominence of low gray values | First order statistics | 10^th^ percentile | 0.03 ± 0.03 | 0.07 ± 0.04 | 0.88 (0.47, 1.28) | |
| *K^trans^* | Prominence of low gray values | GLDM | Low gray level emphasis | 0.21 ± 0.17 | 0.09 ± 0.08 | -0.96 (-1.37, -0.55) | |
| *K^trans^* | Prominence of low gray values | GLRLM | Long run low gray level emphasis | 0.44 ± 0.57 | 0.15 ± 0.23 | -0.75 (-1.15, -0.35) | |
| *K^trans^* | Prominence of low gray values | GLRLM | Low gray level run emphasis | 0.21 ± 0.16 | 0.09 ± 0.08 | -0.96 (-1.37, -0.55) | |
| *K^trans^* | Prominence of low gray values | GLRLM | Short run low gray level emphasis | 0.17 ± 0.11 | 0.08 ± 0.07 | -0.99 (-1.40, -0.58) | |
| *K^trans^* | Prominence of low gray values | GLSZM | Low gray level zone emphasis | 0.19 ± 0.11 | 0.10 ± 0.09 | -0.89 (-1.29, -0.48) | |
|  | | | | | | | |
| *v_e_* | Variance | First order statistics | Robust mean absolute deviation | 0.12 ± 0.06 | 0.07 ± 0.04 | -1.10 (-1.52, -0.69) | |
| *v_e_* | Variance | First order statistics | Interquartile Range | 0.27 ± 0.15 | 0.16 ± 0.08 | -1.02 (-1.44, -0.61) | |
| *v_e_* | Variance | First order statistics | Mean absolute deviation | 0.17 ± 0.06 | 0.11 ± 0.05 | -1.00 (-1.41, -0.59) | |
| *v_e_* | Variance | First order statistics | Variance | 0.05 ± 0.03 | 0.03 ± 0.02 | -0.94 (-1.34, -0.53) | |
| *v_e_* | Variance | First order statistics | 90^th^ percentile | 0.68 ± 0.23 | 0.55 ± 0.23 | -0.61 (-1.00, -0.21) | |
| *v_e_* | Variance | GLCM | Contrast | 108.3 ± 52.4 | 81.2 ± 50.2 | -0.88 (-1.29, -0.48) | |
| *v_e_* | Variance | GLCM | Inverse variance | 0.17 ± 0.06 | 0.24 ± 0.08 | 0.92 (0.51, 1.33) | |
| *v_e_* | Variance | GLCM | Sum squares | 62.8 ± 37.8 | 31.7 ± 27.0 | -0.99 (-1.40, -0.58) | |
| *v_e_* | Variance | GLDM | Gray level variance | 65.8 ± 35.9 | 37.6 ± 28.9 | -0.93 (-1.33, -0.52) | |
| *v_e_* | Variance | GLRLM | Gray level variance | 65.1 ± 36.3 | 36.4 ± 28.2 | -0.92 (-1.32, -0.51) | |
| *v_e_* | Variance | GLSZM | Gray level variance | 64.3 ± 25.8 | 43.2 ± 24.7 | -0.84 (-1.24, -0.43) | |
|  | | | | | | | |
| *v_e_* | Clustering | GLCM | Cluster tendency | 143.0 ± 79.3 | 74.5 ± 60.9 | -0.81 (-1.22, -0.41) | |
| *v_e_* | Clustering | GLCM | Cluster prominence | 82218 ± 67535 | 37127 ± 46922 | -1.00 (-1.42, -0.59) | |
|  | | | | | | | |
| *v_e_* | Heterogeneity | GLCM | Difference average | 7.3 ± 3.1 | 4.8 ± 2.2 | -1.01 (-1.42, -0.59) | |
| *v_e_* | Heterogeneity | GLCM | Difference variance | 40.9 ± 23.8 | 23.5 ± 21.9 | -0.77 (-1.17, -0.37) | |
| *v_e_* | Heterogeneity | GLCM | Id | 0.27 ± 0.07 | 0.33 ± 0.08 | 0.8 (0.39, 1.20) | |
| *v_e_* | Heterogeneity | GLCM | Idm | 0.19 ± 0.06 | 0.24 ± 0.08 | 0.70 (0.30, 1.10) | |
| *v_e_* | Heterogeneity | GLCM | Idmn | 0.93 ± 0.04 | 0.96 ± 0.03 | 0.78 (0.38, 1.18) | |
| *v_e_* | Heterogeneity | GLCM | Idn | 0.85 ± 0.05 | 0.88 ± 0.04 | 0.76 (0.36, 1.16) | |
|  | | | | | | | |
| T2 | Texture complexity | GLCM | Correlation | 0.68 ± 0.15 | 0.76 ± 0.08 | 0.72 (0.32, 1.12) | |
| T2 | Texture complexity | GLSZM | Gray level non uniformity normalized | 0.07 ± 0.05 | 0.05 ± 0.02 | -0.67 (-1.07, -0.27) | |
| T2 | Texture complexity | GLSZM | Zone entropy | 6.16 ± 0.73 | 6.55 ± 0.48 | 0.67 (0.27, 1.07) | |
|  | | | | | | | |
| *v_p_* | Value | First order statistics | Mean | 0.03 ± 0.02 | 0.05 ± 0.03 | 0.75 (0.35, 1.15) | |
| *v_p_* | Value | First order statistics | 90^th^ percentile | 0.05 ± 0.03 | 0.08 ± 0.05 | 0.69 (0.29, 1.09) | |
| *v_p_* | Value | First order statistics | Median | 0.02 ± 0.02 | 0.04 ± 0.03 | 0.74 (0.34, 1.15) | |
| *v_p_* | Value | First order statistics | Root mean squared | 0.04 ± 0.02 | 0.06 ± 0.04 | 0.62 (0.23, 1.02) | |
| *v_p_* | Value | First order statistics | Entropy | 3.06 ± 0.77 | 3.58 ± 0.91 | 0.60 (0.21, 1.00) | |
| *v_p_* | Value | GLCM | Joint average | 5.29 ± 2.98 | 8.66 ± 5.46 | 0.72 (0.31, 1.12) | |
| *v_p_* | Value | GLCM | Sum average | 10.58 ± 5.96 | 17.32 ± 10.92 | 0.72 (0.31, 1.12) | |
| *v_p_* | Value | GLRLM | Run entropy | 3.54 ± 0.66 | 3.98 ± 0.74 | 0.61 (0.21, 1.00) | |
|  | | | | | | | |
| *v_p_* | Prominence of low gray values | First order statistics | 10^th^ percentile | 0.01 ± 0.01 | 0.02 ± 0.01 | 0.84 (0.44, 1.25) | |
| *v_p_* | Prominence of low gray values | GLDM | Low gray level emphasis | 0.24 ± 0.17 | 0.13 ± 0.13 | -0.74 (-1.14, -0.34) | |
| *v_p_* | Prominence of low gray values | GLRLM | Low gray level run emphasis | 0.23 ± 0.15 | 0.13 ± 0.13 | -0.73 (-1.13, -0.32) | |
| *v_p_* | Prominence of low gray values | GLRLM | Short run low gray level emphasis | 0.20 ± 0.12 | 0.12 ± 0.10 | -0.73 (-1.13, -0.33) | |
| Note. Abbreviations: GLCM = gray-level co-occurrence matrix; GLDM = gray-level dependence matrix; GLRLM = gray-level run-length matrix; GLSZM = gray-level size-zone matrix; NGTDM = neighboring gray tone difference matrix. | | | | | | | |
